# Loss of the HPV-Infection Resistance EVER2 Protein Impairs NF-κB Signaling Pathways in Keratinocytes

**DOI:** 10.1371/journal.pone.0089479

**Published:** 2014-02-19

**Authors:** Françoise Vuillier, Guillaume Gaud, Delphine Guillemot, Pierre-Henri Commere, Christian Pons, Michel Favre

**Affiliations:** 1 Unité de Génétique, Papillomavirus et Cancer Humain, Institut Pasteur, Paris, France; 2 Plateforme de cytométrie en flux, Institut Pasteur, Paris, France; University of Birmingham, United Kingdom

## Abstract

Homozygous mutations in *EVER* genes cause epidermodysplasia verruciformis (EV), characterized by an immune defect and the development of skin cancers associated with β- human papillomavirus (HPV) infections. The effects of EVER protein loss on the keratinocyte immune response remain unknown. We show here that EVER2 plays a critical role in the interplay between the NF-κB and JNK/AP-1 signaling pathways. EVER2-deficient cells overproduce IL-6 following the upregulation of JNK activation. They respond poorly to phorbol ester and TNF via the NF-κB pathway. They have lower levels of IKKα subunit, potentially accounting for impairments of p100 processing and the alternative NF-κB pathway. The loss of EVER2 is associated with an unusual TRAF protein profile. We demonstrate that EVER2 deficiency sustains TRAF2 ubiquitination and decreases the pool of TRAF2 available in the detergent-soluble fraction of the cell. Finally, we demonstrate that EVER2 loss induces constitutive PKCα-dependent c-jun phosphorylation and facilitates activation of the HPV5 long control region through a JNK-dependent pathway. These findings indicate that defects of the EVER2 gene may create an environment conducive to HPV replication and the persistence of lesions with the potential to develop into skin cancer.

## Introduction

Cutaneous human papillomavirus (HPV) can induce diverse skin lesions, from warts to fully invasive carcinomas [1], [2]. The host genetic factors favouring the malignant transformation of oncogenic HPV-infected keratinocytes have not been fully elucidated. The first reported evidence for this association was obtained from patients with epidermodysplasia verruciformis (EV) [3]. This rare autosomal recessive skin disease (OMIM 226400) is associated with abnormally high susceptibility to β-HPVs. EV patients have disseminated skin lesions and frequently develop squamous cell carcinoma induced by HPV5 and 8 [1], [4]. They have defective cell-mediated immunity, resulting in the persistence of lesions and high loads of the infecting β-HPVs [2]. EV thus constitutes a model of genetic skin cancer induced by specific HPVs [3].

We have demonstrated that homozygous mutations of *EVER1* (*TMC6*) and *EVER2* (*TMC8*) are associated with EV [5]. These genes are expressed in hematopoietic and endothelial cells [6], consistent with a role in immunity to HPV [2]. The EVER proteins are probably involved in signal transduction pathways in keratinocytes, but their exact role remains unclear. They may regulate zinc homeostasis and the activity of the transcription factor AP-1, a key element in the HPV life cycle [7].

Many studies have provided evidence of a link between NF-κB activation and hematological and epithelial cancers [8], [9]. There is also evidence to suggest that NF-κB inhibition increases the incidence of liver and skin cancer, implying cell-specific effects [9], [10], [11]. The over-production of TNF and TGF-β in the lesional epidermis of EV patients [4] suggested a possible role for inflammatory responses in regulating the growth and differentiation of HPV-infected keratinocytes and in lesion persistence. EVER proteins may thus be involved in regulating the NF-κB and JNK/AP1 pathways and may contribute to HPV-induced carcinogenesis in keratinocytes. We investigated the molecular processes underlying the cancer progression associated with β-HPV infection in the genetic disorder of EV, by assessing the impact of EVER2 loss on the NF-κB and JNK activation pathways, using wild-type and EVER2-deficient keratinocytes. We found that EVER2 loss induced constitutive JNK activation, promoting HPV5 LCR activation and inflammatory responses and highlighting the crucial role of EVER2 in control of the NF-κB and JNK/AP-1 signaling pathways.

## Results and Discussion

### EVER2 deficiency is associated with an abnormal pattern of IL-6 production

**Figure 1:**
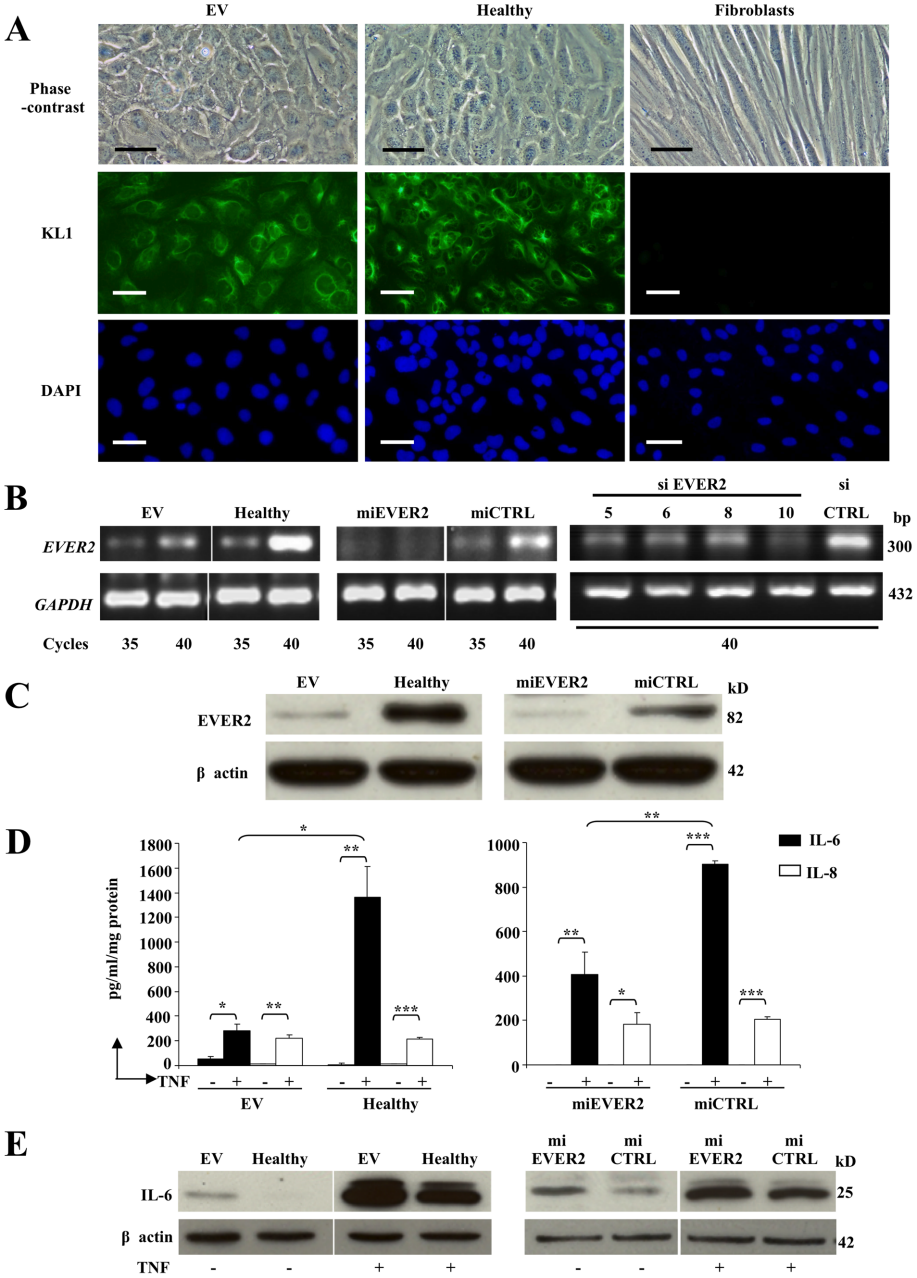
Characterization of cell lines. (A) EV and Healthy cell lines were compared with fibroblasts. They were studied by phase-contrast microscopy. Bars, 50 µm. They were also stained for cytokeratin (KL1); the nucleus was stained with DAPI. Bars, 100 µm. (B–C) *EVER2* expression was determined in EVER2^−/−^ cell lines (EV and miEVER2) compared with control cell lines (Healthy and miCTRL). *EVER2* transcripts (B) were studied by semiquantitative RT-PCR. Healthy cells transfected with control siRNA (siCTRL) were also compared with cells transfected with siRNAs targeting various exons (5, 6, 8 and 10) of *EVER2* (siEVER2). The levels of transcripts after various numbers of cycles are shown for RT-PCR studies. *GAPDH* transcripts were used as the reference. The production of EVER2 protein (C) was evaluated by the western blotting of insoluble fractions isolated from the cells. The data shown are representative of three independent experiments. (D–E) IL-6 production was studied in EVER2^−/−^ (EV and miEVER2) and wild-type (Healthy or miCTRL) cells. The cells were cultured in the presence or absence of TNF. (D) After 48 h, the supernatants were collected and tested for IL-6 and IL-8 by flow cytometry. Data are means ± SD of three independent experiments. (E) After 16 h in the presence of brefeldin A, whole-cell lysates were assayed for intracellular IL-6 by western blotting. Data are from one experiment representative of three independent experiments carried out. *, *P*<0.05; **, *P*<0.01; ***, *P*<0.001.

We investigated the possible functional consequences of the homozygous truncating mutations of *EVER*2 causing EV [5], in keratinocyte cell lines lacking EVER2. We generated EVER2^−/−^ keratinocyte cell lines from an EV patient (EV cell line), and from a healthy subject, by silencing *EVER2* expression with microRNA (miEVER2 cell line). Wild-type *EVER2* cell lines were generated from a healthy subject and are referred to as the “Healthy cell line” and the “miCTRL cell line” if transfected with control miRNA. All cell lines had a keratinocyte phenotype. In our culture conditions, they kept a squamous epithelial cell character as shown by polygonal figures in phase-contrast microscopy (Fig. 1A). We confirmed also that the cells we are using are keratinocytes by immunofluorescence experiments with pan-cytokeratin antibody (KL1) (Fig. 1A). We observed an intense labelling of the cells in EV and Healthy cell lines as opposed to fibroblasts where no labelling was observed. EVER2 knockdown was confirmed in EVER2^−/−^ cells by semi-quantitative RT-PCR (Fig. 1B) and by western blotting (Fig. 1C). A very low amount of EVER2 protein was still detected in EV cells (Fig. 1C). We cannot exclude the possibility that EV cells produce a truncated and non functional EVER2 protein which is a little smaller than the full-length following a deletion of the premature stop codon in exon 5.

We first analyzed the characteristics of EVER2^−/−^ cells in terms of cytokine secretion in response to stimulation with TNF. We assayed IL-6, IL-8, IL-10, IFN-γ, IL-2, IL-5, IL-4, IL-1β and IL-12; only IL-6 and IL-8 were detected in supernatants after stimulation with TNF. These two cytokines were present at levels only just above the detection threshold in the absence of stimulation (Fig. 1D). IL-6 concentrations in the supernatants of EVER2^−/−^ cell lines (EV or miEVER2) were lower than those of controls (Healthy or miCTRL). By contrast, IL-8 concentrations did not differ between EVER2^−/−^ cell lines and controls (Fig. 1D). We then used western blotting to assess IL-6 production by EVER2-deficient cells treated with brefeldin A to block secretion. The EVER2^−/−^ cell lines constitutively produced more IL-6 than the controls (Fig. 1E). In addition, TNF increased the intracellular production of IL-6 to a greater extent in both EVER2^−/−^ cell lines than in controls (Fig. 1E). Thus, EVER2 loss results in the enhanced intracellular production of IL-6, which may contribute to the formation of an inflammatory microenvironment facilitating cancer development [12]. However, the mechanisms underlying the lower free IL-6 concentration in the supernatants of EVER2^−/−^ cell cultures remain unclear: this phenomenon may involve an impairment of IL-6 secretion, the retention of IL-6 on IL-6 receptors, a shorter half-life or a folding defect of the cytokine itself.

### EVER2-deficient cells respond poorly to TNF and phorbol ester

**Figure 2:**
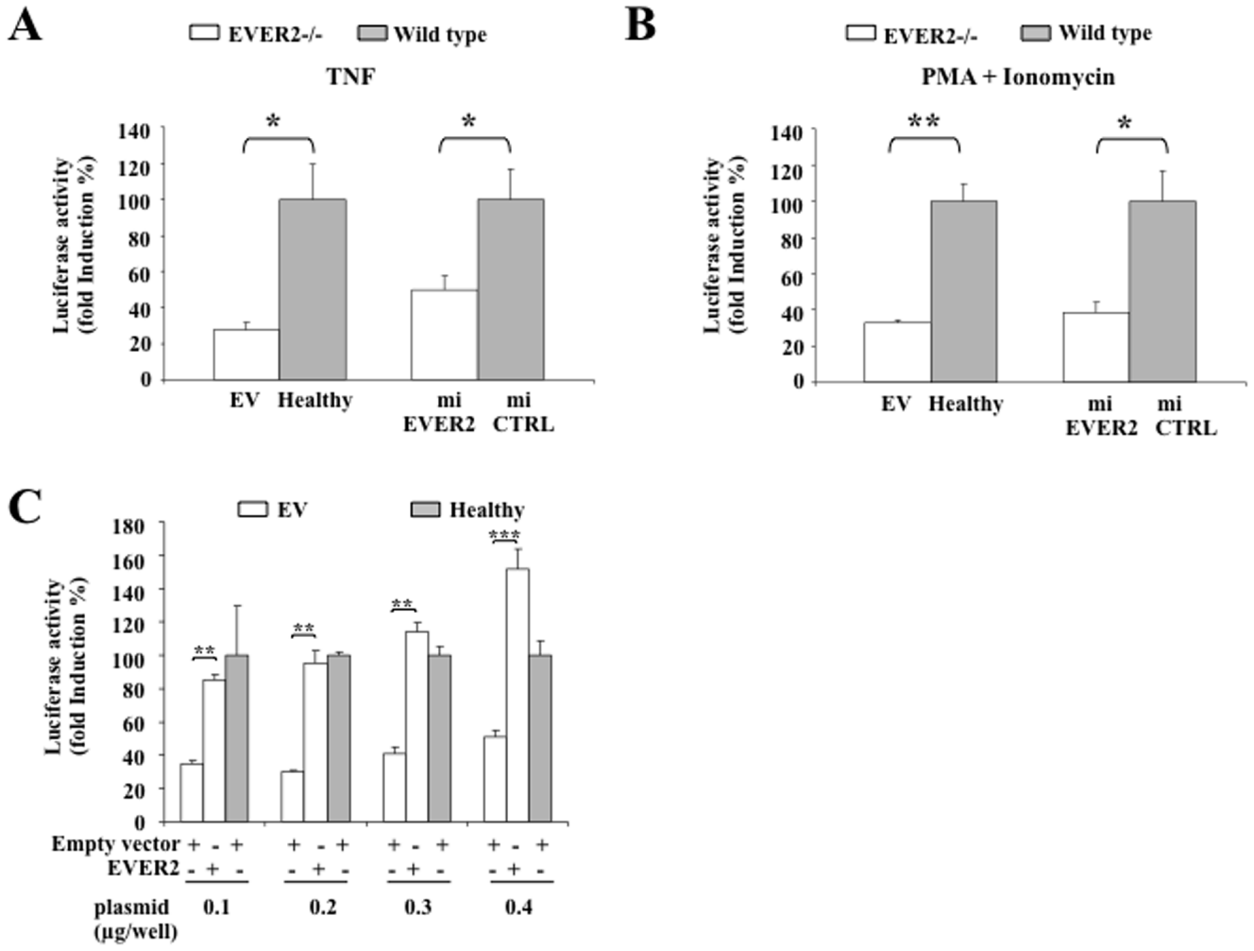
EVER2 regulates the transcriptional activity of NF-κB. (A–B) Keratinocyte cell lines were transiently transfected with 0.3 µg/well NF-κB luciferase reporter. The cells were serum-starved overnight and left untreated or were stimulated with TNF (A) or a combination of PMA+ionomycin (B). The cells were harvested and assayed for luciferase activity. The data shown are expressed as fold-induction with respect to nonstimulated cells. Values obtained with wild type cells were taken as 100%. The data shown are means ± SD of three independent experiments. (C) EV or healthy cells were transiently transfected with the NF-κB/luc construct (0.3 µg/well) and various amounts of a plasmid encoding EVER2 or an empty vector as a control. Cells were assayed for luciferase activity after stimulation with TNF following serum starvation. The data shown are expressed as fold-induction with respect to nonstimulated cells. Values obtained with healthy cells transfected with empty vector were taken as 100%. The data shown are means ± SD of three independent experiments. *, *P*<0.05; **, *P*<0.01; ***, *P*<0.001.

The involvement of NF-κB in the production of cytokines, such as IL-6 and TNF, and in cancer-related inflammation is well established [8], [12]. We investigated the effect of EVER2 deficiency on NF-κB activity. Keratinocyte cell lines were transiently transfected with the NF-κB/luc construct and were left untreated or stimulated with TNF. Lower levels of fold-induction in response to TNF were observed in EVER2-deficient cells than in control cells (Fig. 2A). A similar downregulation of NF-κB activity in EVER2^−/−^ cells was observed after stimulation with a combination of phorbol myristate acetate (PMA) and ionomycin (Fig. 2B). We also tried to restore the wild-type phenotype in EVER2^−/−^ cells. We were unable to generate stable EV cell lines reconstituted with wild-type EVER2 because the sustained overexpression of *EVER2* led to cell death [13]. EV cells reconstituted with EVER2 by transient transfection regained the ability to upregulate NF-κB activity following TNF treatment (Fig. 2C). These findings confirm that EVER2-deficient cells respond poorly to TNF. The transfection of these cells with *EVER2* cDNA restores the wild-type phenotype, suggesting that EVER2 may control NF-κB signaling pathways and act as a critical modulator of the inflammatory response in keratinocytes.

### EVER2 deficiency impairs NF-κB signaling pathways

**Figure 3:**
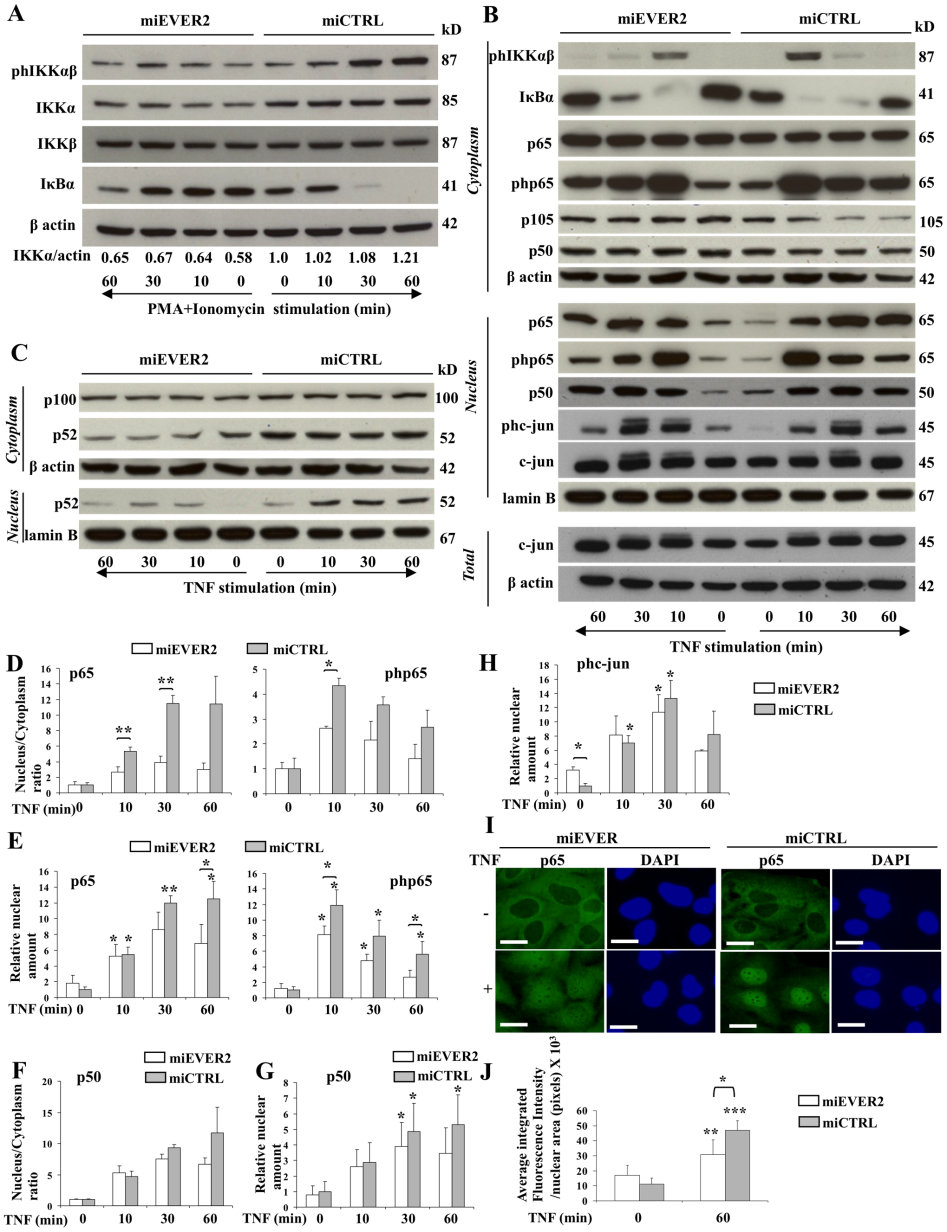
Function of EVER2 in NF-κB and c-jun activation. miEVER2 and miCTRL cell lines were left untreated or were stimulated with PMA+ionomycin (A) or TNF (B–J) for the times indicated. (A) Whole-cell lysates were analyzed by western blotting to explore the IKKαβ complex. The phosphorylated forms of IKKαβ are indicated as phIKKαβ. The IKKα and actin bands on western blots were quantified by densitometry. Results are reported as the ratio of IKKα to actin. The ratio obtained for the miCTRL cell line at time 0 was set to 1. (B–C) Cytoplasmic and nuclear fractions were analyzed for the classical NF-κB and AP-1 axis (B) or for the alternative NF-κB axis (C). The phosphorylated forms of p65 and c-jun are indicated as php65, and phc-jun respectively. Whole-cell lysates (Total) were analyzed for total c-jun expression. (D–H) Bands on western blots were quantified by densitometry. (D–E) The amounts of p65 and phosphorylated p65 (php65) were quantified in nuclear and cytoplasmic fractions, following stimulation with TNF. The results are reported as the ratio of nuclear and cytoplasmic concentrations (D). The ratios for basal levels were set to 1. (E) The amounts of p65 and php65 in nuclear fractions are reported. Basal levels of miCTRL were set to 1. (F–H) Subcellular extracts were assayed for p50 and the phosphorylated form of c-jun (phc-jun). For p50, the results are reported as the ratio of nuclear and cytoplasmic concentrations (F). Ratios for basal levels were set to 1. Total amounts of p50 (G) and phosphorylated c-jun (phc-jun) (H) in the nuclear fraction of cells are reported. Basal levels of miCTRL were set to 1. Data are means ± SD of three independent experiments. Statistically significant differences between the results for two cell lines are indicated by asterisks over the brackets. Asterisks over error bars indicate statistically significant differences in the results between unstimulated and TNF-stimulated cells. (I–J) Keratinocytes from the miEVER or the miCTRL cell lines were left untreated or were treated with TNF for 1 hour and stained for NF-κB (p65); the nucleus was stained with DAPI (I). Bars, 20 µm. (J) The fluorescence intensity of nuclear p65 was quantified before and after stimulation. Asterisks over error bars indicate statistically significant differences in fluorescence intensity between unstimulated and TNF-stimulated cells. Statistically significant differences between the two cell lines are indicated by asterisks over the brackets. *, *P*<0.05; **, *P*<0.01; ***, *P*<0.001.

TNF and PMA+ionomycin use different upstream pathways converging on IKK activation to stimulate NF-κB [14] (Fig. S1). The changes in luciferase activity in response to these two stimuli led us to assess phosphorylation of the IKK complex upon stimulation. IKKαβ complex phosphorylation levels were lower in EVER2^−/−^ cells than in controls following stimulation with PMA+ionomycin (Fig. 3A and S2A). Furthermore, IKKαβ phosphorylation was found to be defective following TNF stimulation in EVER2^−/−^ cells (Fig. 3B and S2B). These results indicate that EVER2 deficiency impairs NF-κB signaling pathways.

The abnormal production of IKKα and IKKβ is associated with skin inflammation, and poor IKKα production has been reported to promote skin tumor development [15], [16], [17], [18]. We therefore investigated whether EVER2 controlled the levels of IKKα and β subunits, by assessing the amounts of IKKα and β in our cell lines. Unlike IKKβ, IKKα was less abundant in EVER2^−/−^ cells than in control cells, even after PMA+ionomycin stimulation (Fig. 3A and S2A). Thus, EVER2 deficiency impairs the IKKαβ complex by decreasing the amount of IKKα.

Previous studies have suggested that the most important function of IKKα is the control of p100 processing, leading to activation of the alternative pathway [19] (Fig. S1). We therefore investigated the effects of EVER2 knockdown on the processing of p100 to p52. Constitutively produced p52 was less abundant in the cytoplasm and nucleus of EVER2^−/−^ cells than in those of control cells (Fig. 3C and S2C). After exposure to TNF, p52 levels remained lower in both compartments of EVER2^−/−^ cells than in those of control cells. A defect in basal p52 production was confirmed in the nucleus of healthy cells treated with siRNAs targeting exons 5, 6, 8 and 10 of the *EVER2* gene (Fig. S3B). Consistent with the low levels of IKKα, these findings indicate that EVER2 loss prevents p100 processing, thereby impairing the alternative NF-κB signaling pathway.

The IKKβ subunit is involved principally in activation of the classical NF-κB pathway and it can replace IKKα if this subunit is missing [16]. We then investigated the effect of EVER2 deficiency on the classical NF-κB signaling axis, by assessing IκBα levels before and after exposure to PMA+ionomycin or TNF (Fig. S1). No IκBα degradation was detected in EVER2^−/−^ cells in the basal state, and that following both stimulations was less marked than in control cells (Fig. 3A, 3B, S2A and S2B). As p65 translocation may occur without IκBα degradation [20], we assayed the p65 subunit in the cytoplasmic and nuclear fractions of all cell lines by western blotting before and after stimulation with TNF only (Fig. S1, 3B and S2B), and assessed the p65 translocation by determining the nuclear to cytoplasmic ratio (as illustrated for miEVER2 cell line; Fig. 3D). The amounts of p65 and its phosphorylated form (php65) translocated into the nucleus were smaller in miEVER2 cells than in controls (Fig. 3D). This translocation defect was confirmed by quantifying p65 and php65 in nuclear extracts from miEVER2 cells and controls (Fig. 3E). Nevertheless, the translocation defect occurred shortly after stimulation (from 10 min onwards) (Fig. 3D). After stimulation with TNF for 1 hour, the amounts of p65 and its phosphorylated form in the nucleus were significantly lower in EVER2^−/−^ cells than in control cells (Fig. 3E). Immunofluorescence experiments confirmed a translocation defect of p65 into the nucleus in EVER2^−/−^ cells following TNF stimulation. We observed a diffuse staining in cytoplasmic and nuclear compartments of EVER2^−/−^ cells as opposed to control cells where an intense labelling of nuclei was observed (Fig. 3I and S2D). Fluorescence microscopy analysis confirmed also that the p65 subunit was less abundant in the nucleus following TNF stimulation in EVER2^−/−^ cells than in control cells (Fig. 3J and S2E).

No significant difference was found between EVER2^−/−^ cells and controls in terms of the translocation of p50 or the amount of this protein in the nucleus, at any time point after TNF stimulation (Fig. 3F and 3G). However, TNF induced no significant increase in the amount of p50 over the basal level in unstimulated cells after the stimulation of EVER2^−/−^ cells for 1 h, by contrast to the findings for normal cells (Fig. 3G).

Consistent with the data presented in figure 2A, these findings indicate that EVER2 deficiency impairs the classical and alternative pathways following exposure to TNF.

TNF is also a potent activator of c-Jun N-terminal kinase (JNK), which regulates the activation of AP-1 transcription factors, such as c-jun [21], [22] (Fig. S1). We investigated the role of EVER2 in the interplay between TNF-mediated signaling pathways, by assessing the effect of EVER2 knockdown on the AP-1 pathway through evaluation of the phosphorylation of c-jun in the nucleus. Basal c-jun phosphorylation was more extensive in EVER2^−/−^ cells than in control cells (Fig. 3B, 3H and S2B). Following TNF stimulation, EVER2^−/−^ cells responded slightly less strongly than normal cells via the JNK signaling pathway (Fig. 3H). However, no significant differences in the amount of phosphorylated c-jun in the nucleus were found between the two cell lines, at any time point after TNF stimulation (Fig. 3H). Basal c-jun phosphorylation was confirmed in healthy cells treated with siRNAs targeting parts 5, 6, 8 and 10 of the *EVER2* gene (Fig. S3B). We also assessed the total amounts of c-jun in nuclear and whole-cell extracts of EVER2^−/−^ and control cells. EVER2 knockdown had no effect on total c-jun levels (Fig. 3B and S2B). Only cells treated with an siRNA against exon 8 showed an increase of total c-jun levels (Fig. S3B).

Collectively, these results support the notion that EVER2 prevents constitutive JNK activation but targets upstream signaling molecules, to provide an immediate and efficient response to stimulation with TNF or PMA+ionomycin, a potent activator of PKC, through NF-κB signaling pathways.

### EVER2 deficiency impairs TRAF protein production and sustains TRAF2 ubiquitination

**Figure 4:**
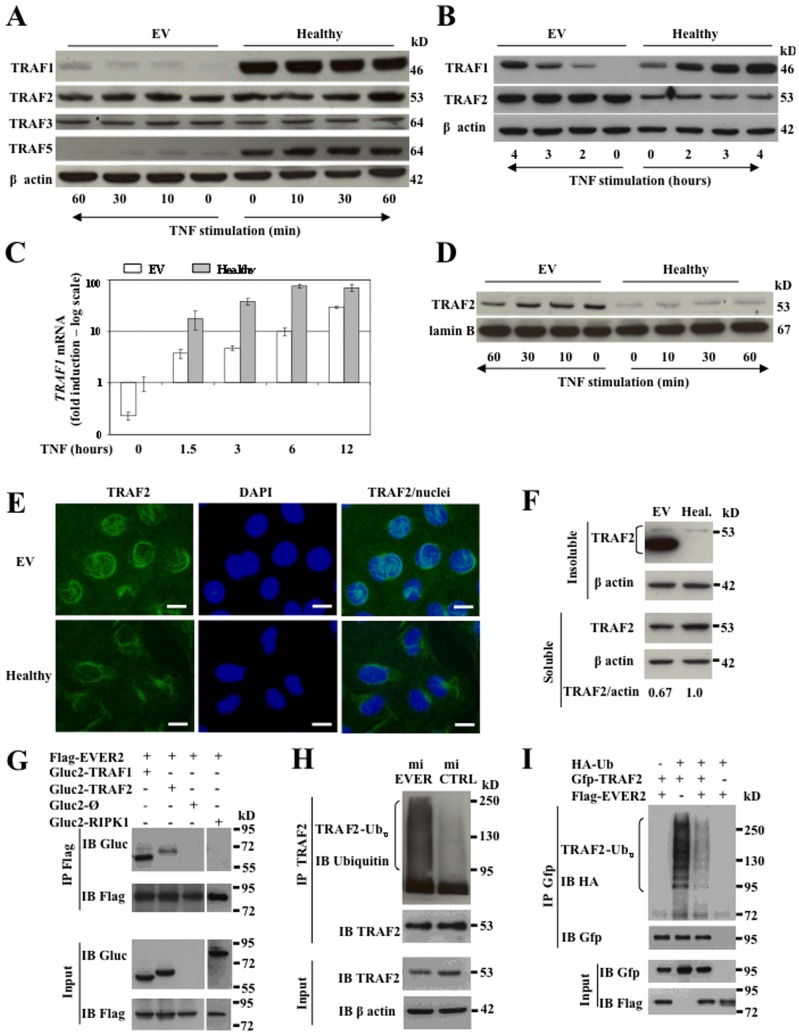
EVER2 deficiency impairs TRAF protein production. (A–D) EV and Healthy cell lines were left untreated or stimulated with TNF for the time periods indicated. (A) Cytoplasmic extracts were subjected to western blotting. The results shown are representative of three independent experiments. (B) Whole-cell lysates were analyzed by western blotting after various times. (C) qRT-PCR was used to measure the induction of *TRAF1* 1.5–12 h after stimulation with TNF. Results are reported as fold-induction with respect to untreated cells. The basal level for the Healthy cell line was set to 1. A logarithmic (log) scale was used for the *Y* axis. Experiments were performed twice independently. (D) Nuclear extracts were subjected to immunoblot analysis with TRAF2 antibody for the times indicated. The results shown are representative of three independent experiments. (E–F) EV and Healthy (Heal.) cell lines were left untreated. (E) Cells were stained for TRAF2; the nucleus was stained with DAPI. Bars, 20 µm. (F) Keratinocytes were lysed in Triton X-100 lysis buffer. Soluble and insoluble fractions were subjected to immunoblot analysis with TRAF2 antibody. The TRAF2 and actin bands were quantified by densitometry for the soluble fraction. Results are reported as the ratio of TRAF2 to actin. The ratio obtained for the Healthy cell line was set to 1. (G) HEK-293T cells were transfected with plasmids encoding Flag-tagged EVER2 and Gluc2-tagged TRAF1, TRAF2 or RIPK1 proteins or Gluc2 fragment as a control (Gluc2-Ø). Crude lysates were immunoprecipitated with anti-Flag antibody (IP) and immunoblotted (IB) with the antibodies indicated. (H) Endogenous TRAF2 was immunoprecipitated (IP) with anti-TRAF2 in miEVER2 and control cells, and then subjected to immunoblotting (IB) with anti-Ubiquitin (Ub) antibody. The results shown are representative of three independent experiments. (I) HEK-293T cells were cotransfected with Gfp-tagged TRAF2, hemaglutinin (HA)-tagged Ubiquitin (Ub) with or without Flag-EVER2. After 24 h, the cells were treated with TNF for 2 h. Lysates were subjected to immunoprecipitation and immunoblotting with the indicated antibodies. The results shown are representative of two independent experiments.

TNF receptor-associated factors (TRAF) play a major role in linking the TNF receptor to downstream signaling pathways [23]. However, TRAF2 has also been reported to be essential for PKC-dependent NF-κB signaling and JNK activation [24], [25], [26], [27]. Changes in expression level or intracellular localization have been identified as important regulatory mechanisms for TRAF-mediated signal transduction [23], [28]. We therefore investigated whether EVER2 deficiency affected levels of TRAF1, 2, 3 and 5. On western blots, TRAF1 and TRAF5 were less abundant in cytoplasmic extracts from EV cells than in those from control cells (Fig. 4A). By contrast, no significant differences were found in the amounts of TRAF2 and TRAF3. Similarly, miEVER2 cells contained less TRAF1 than controls, but no difference in TRAF5 levels was observed (Fig. S3A). In normal cells, defects in TRAF1 and TRAF5 expression were observed following the silencing of EVER2 with siRNAs targeting the exons 5, 6, 8 and 10 of this gene (Fig. S3B). We then assessed the capacity of the EV cell line to produce TRAF1, which can be induced with TNF [29]. Total TRAF1 and TRAF1 mRNAs were less abundant, before and after TNF induction, in EVER2^−/−^ cells than in control cells (Fig. 4B and 4C). This defect may reflect deregulation of NF-κB signaling pathway. Nevertheless, additional mechanisms may contribute to the sharp loss of TRAF1 protein, including TGF-β production [30], caspase-dependent cleavage [31], proteosome-dependent degradation, mRNA instability or a short half-life of TRAF1 protein. However, negative results were obtained in all the experiments carried out (data not shown). Our data clearly show that the loss of TRAF1 is EVER2-dependent, but the exact mechanism involved is unknown.

TRAF1 works in conjunction with TRAF2 and may regulate TRAF2 localization, which is crucial for the activation of downstream signaling cascades [26], [28]. Given the major TRAF1 deficiency, we checked for a possible abnormal localization of TRAF2 in EV cells with the lowest level of TRAF1 expression. Western blotting revealed no abnormality in cytoplasmic extracts (Fig. 4A), but there was more TRAF2 in whole-cell extracts from EV cells than in control cell extracts (Fig. 4B). This difference reflected the larger amounts of TRAF2 in nuclear extracts from EV cells than in those from control cells (Fig. 4D). Immunofluorescence experiments confirmed the abnormal localization and abundance of TRAF2 in EV cells compared with controls (Fig. 4E). The presence of larger amounts of TRAF2 in nuclear extracts was also confirmed in normal cells after the silencing of EVER2 with siRNAs specifically targeting exons 5 and 10 (Fig. S3B). We then investigated whether EVER2 loss affected the level of soluble TRAF2 required for the subsequent stimulation by TNF by assessing the amounts of TRAF2 in Triton-soluble and -insoluble fractions of cells at steady state. EV cells had larger amounts of TRAF2 in insoluble fractions than the control cells (Fig. 4F). This abnormal localization was associated with a reduction of TRAF2 in detergent-soluble fraction. In addition, in the insoluble fraction of EV cells, TRAF2 appeared to be a doublet, with one form migrating more rapidly. This may ultimately lead to degradation of TRAF2 [28]. Thus, EVER2 loss induced an accumulation of TRAF2 in detergent-insoluble fraction and reduced the pool of TRAF2 available in the soluble fraction. We therefore investigated whether EVER2 could interact with TRAF1 or TRAF2, regulating the functions of these proteins. Co-immunoprecipitation experiments showed that EVER2 formed a complex with both TRAF1 and TRAF2 (Fig. 4G). By contrast, no complex was detected with the receptor-interacting serine/threonine-protein kinase 1 (RIPK1) confirming the specificity of the binding to EVER2 (Fig. 4G).

The polyubiquitination of TRAF2, a downstream target of PKC, has been shown to contribute to IKK and JNK activation [24]. We therefore investigated whether EVER2 affected TRAF2 ubiquitination. TRAF2 was constitutively ubiquitinated in miEVER2^−/−^ but not in control cells (Fig. 4H). For confirmation of the effect of EVER2 on TRAF2 ubiquitination, we performed TRAF2 immunoprecipitation assays in HEK-293T cells following the overproduction of EVER2 together with TRAF2 and ubiquitin. The overproduction of EVER2 and TRAF2 resulted in much lower levels of the ubiquitinated forms of TRAF2 following TNF stimulation (Fig. 4I). Thus, EVER2 loss sustains constitutive TRAF2 ubiquitination consistent with basal JNK activation [24], [27]. This event may, in turn, keep TRAF2 insoluble, thereby decreasing the pool of soluble TRAF2 available for subsequent stimulation by TNF or PMA+ionomycin [28]. This suggests that the relocalization of TRAF2 is defective in EVER2-deficient cells. However, we cannot exclude the possibility that TRAF1 loss contributes to this defect, as previously reported [28], [32].

### EVER2 deficiency drives an inflammatory response by promoting JNK activation and sustains the PKCα-dependent phosphorylation of c-jun

**Figure 5:**
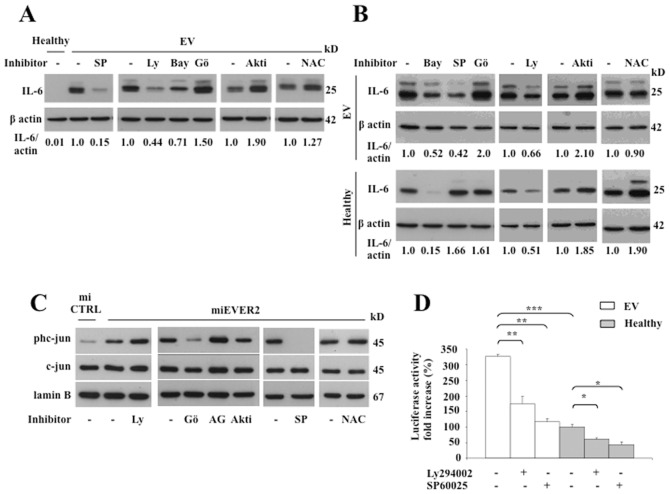
EVER2 controls NF-κB and JNK/AP-1 pathways. (A–D) Cell lines were left untreated (-) or were treated with specific inhibitors of JNK (SP60025 (SP; 20 µM)), PI3K (Ly294002 (Ly; 15 µM)), IKKβ (Bay11-7082 (Bay; 5 µM)), PKCα (Gö6976 (Gö; 0.5 µM)), AKT1/2 (Akti; 2 µM), EGF receptor (AG1478 (AG; 12.5 µM) or N-acetylcysteine (NAC; 10 µM) as indicated. EV and Healthy cell lines were left unstimulated (A) or were stimulated with TNF (B) in the presence of brefeldin A for 16 hours. Whole-cell lysates were assayed for intracellular IL-6 by western blotting. The data shown are from one experiment representative of two independent experiments carried out. The IL-6 and actin bands on western blots were quantified by densitometry. Results are reported as the ratio of IL-6 to actin. In panel A, the ratio obtained for untreated EV cells was set to 1. In panel B, the ratio was set to 1 for each cell line in the absence of inhibitors. (C) miEVER2 and miCTRL cell lines were left untreated (-) or were treated with inhibitors specific as indicated. We analyzed the levels of c-jun or its phosphorylated form (phc-jun) in nuclear extracts by western blotting. The results shown are representative of two independent experiments. (D) EV and Healthy cell lines were transiently transfected with 0.3 µg/well HPV5-flLCR/luc or HPV5-minLCR/luc. Cells were assayed for luciferase activity. Results are reported as fold-increases over the level obtained with HPV5-minLCR/luc. Values obtained with the unstimulated Healthy cell line are set to 100%. The data shown are means ± SD of three independent experiments. *, *P*<0.05; **, *P*<0.01; ***, *P*<0.001.

We observed an unusual amount of IKKα at the basal level (Fig. 3A time 0 and Fig. S2A time 0) associated with a defective response to TNF through NF-κB pathways in EVER2^−/−^ cells (Fig. 3 panels B to G, I and J and S2 panels B to E). We thus carried out inhibition assays, to elucidate the signaling pathways involved in IL-6 production in EVER2-deficient cells (Fig. 1E). Constitutive and TNF-induced IL-6 production in EV cells was decreased by SP60025 and, to a lesser extent, by Bay11-7082 and Ly294002, specific inhibitors of JNK, IKKβ and PI3K, respectively (Fig. 5A and 5B top panel). By contrast, no basal IL-6 production was detectable in the Healthy cell line (Fig. 1E and 5A), in which TNF-induced IL-6 production was largely inhibited by treatment with Bay11-7082 and to a lesser extent by Ly294002 (Fig. 5B bottom panel). The scavenging of reactive oxygen species (ROS) with N-acetylcysteine (NAC) had no effect on IL-6 levels in EV cell line, ruling out the involvement of ROS production in this process (Fig. 5A and 5B). Thus, JNK activation contributed to the higher levels of IL-6 production in EVER2-deficient cells. This overproduction may involve a cooperative interaction between NF-κB and AP-1 sites in the IL-6 promoter or a JNK positive feedback amplification loop [33]. These findings demonstrated that PI3K and JNK activation was required to induce inflammation in EVER2-deficient cells, a process that was counteracted by an AKT-dependent signaling as shown by the increase of IL-6 production following treatment with Akt1/2 inhibitor (Fig. 5A).

We next investigated the kinases involved in the constitutive nuclear phosphorylation of c-jun in EVER2^−/−^ cells. This phosphorylation was largely inhibited by Gö6976, a PKCα inhibitor, and was completely abolished by SP60025, a JNK inhibitor (Fig. 5C). By contrast, no change was detected with ROS scavengers, such as NAC. Activation of the epidermal growth factor (EGF) receptor was not involved in the phosphorylation process, because specific inhibitors of this receptor did not prevent c-jun phosphorylation. Thus, EVER2 loss induced high basal levels of c-jun phosphorylation through a PKCα/JNK-dependent pathway.

### The loss of EVER2 facilitates HPV5 LCR activation via the PI3K and JNK signaling pathways

The activation of AP-1 transcription factors, such as c-jun, is important for β-HPV transcription [34]. As basal levels of c-jun phosphorylation were higher in EVER2-deficient cells than in control cells, we hypothesized that the AP-1 pathway might influence HPV5 LCR activation in EVER2^−/−^ cells. We tested this hypothesis, by analyzing the expression of a luciferase reporter gene under the control of a full-length LCR from HPV5 (HPV5-flLCR/luc) containing AP-1-responsive elements. Luciferase activity levels were higher in the EV cell line than in controls (Fig. 5D). Similar results were obtained for the miEVER2 cell line (data not shown). The inhibition of PI3K and JNK by Ly294002 and SP60025, respectively, decreased luciferase activity more strongly in EVER2^−/−^ cell lines than in controls (Fig. 5D). These data are consistent with constitutive activation of the JNK/AP-1 pathway in EVER2-deficient cells and show that EVER2 loss facilitates HPV5 LCR activation via the PI3K and JNK signaling pathways.

Overall, we demonstrate here that EVER2 loss promotes a PI3K- and JNK-dependent activation pathway, increasing HPV5 LCR activation and IL-6 production. By contrast, the NF-κB response to PMA+ionomycin was defective in EVER2^−/−^ cells. We also found that EVER2-deficient cells responded poorly to TNF through impaired alternative and classical NF-κB pathways. All these findings suggest that EVER2 prevents PKCα-dependent JNK activation and targets TRAF2 through effects on its ubiquitination and localization, leading to efficient responses to TNF through the NF-κB and JNK/AP-1 signaling pathways.

Thus, our findings indicate that a genetic defect may create a microenvironment facilitating HPV transcription but preventing the NF-κB response to subsequent inflammatory challenge. This is one possible explanation for both the persistence of lesions and the low invasive and metastatic potential of EV cancers.

## Materials and Methods

### Cell lines and culture conditions

Keratinocytes were obtained from hair follicles from a healthy subject and from a Polish EV patient carrying a homozygous mutation characterized by a splice site mutation IS4-1G>T, T150fsX3 mutation in the *EVER2* gene [7] as described by Limat A. and F.K. Noser [35]. They were cultured on a feeder layer of lethally irradiated mouse 3T3-J2 fibroblasts [36]. Keratinocytes were immortalized with two lentiviruses encoding telomerase and SV40-AgT [37]. Immortalized cells were maintained in culture for four months in K-SFM medium (Gibco) without a feeder layer. The immortalized keratinocyte cell lines were then allowed to adapt to growth in serum-containing medium. The study was approved by the Ethics Committee at the Medical University of Warsaw. Written informed consent was received from all participants.

For EVER2 silencing, a microRNA expression vector specific for EVER2 (miEVER2) was generated with the BLOCK-iT™ Pol II miR RNAi Expression Vector Kit (Invitrogen) according to the manufacturer's instructions. Two single-stranded DNA oligonucleotides encoding the pre-miRNA were designed with RNAi Designer (Invitrogen), according to the manufacturer's instructions. These oligonucleotides targeted the following sequence of the *EVER2* gene (encoding part of the N-terminal region of the protein): 5′-CAGGAATTCGGTCCTACTTCA-3′. Cell lines from a healthy subject were transfected with miEVER2 or a control (miCTRL) expression vector containing an EmGFP-coding sequence in the presence of Lipofectamine 2000 reagent (Invitrogen) according to the manufacturer's instructions. The cells expressing the pre-miEVER2 and control constructs were positive for green fluorescence protein (GFP) and were purified by cell sorting with a MoFLO flow cytometer (Beckman Coulter, Villepinte, France). All keratinocyte cell lines were grown in MEM containing 10% fetal calf serum, penicillin/streptomycin and 2 mM L-glutamin.

In some experiments, cells were transfected with pre-designed siRNAs (Applied Biosystems) directed against different parts (exons 5, 6, 8 or 10) of *EVER2* (siEVER2) or control siRNAs (siCTRL). *EVER2* transcripts levels were then assessed by semi-quantitative RT-PCR (Fig. 1B).

For all stimulation experiments, cells were serum-starved overnight and treated with 10 ng/ml recombinant human TNFα (TNF) or a combination of 10 ng/ml PMA plus 50 ng/ml ionomycin, for various periods of time.

HEK-293T cells were cultured in DMEM containing 10% fetal calf serum, penicillin/streptomycin and 2 mM L-glutamin.

### Antibodies and reagents

Antibodies against TRAF1, TRAF2, TRAF3, TRAF6, p50, p52, p105, p100, IκBα, the phosphorylated forms of IKKα (Ser176) β (Ser177), p65 (Ser536), c-jun (Ser63) and IL-6 were purchased from Cell Signaling Technology. Antibodies against p65 and TRAF5 were obtained from Santa Cruz Biotechnology. The antibody against EVER2 was purchased from Interchim. The anti-lamin B antibody was obtained from Thermo Scientific. Recombinant human TNFα was purchased from Miltenyi Biotec. Anti-actin, anti-Flag and anti-hemaglutinin (HA) antibodies, PMA, ionomycin, brefeldin A, Ly294002, SP60025, Bay 11-7082, Gö6976, AG1478, Akt1/2 kinase inhibitor and N-acetylcysteine were obtained from Sigma-Aldrich. Anti-Gluc and anti-Gfp antibodies were obtained from BioLabs. Anti-KL1 antibody was purchased from Immunotech Biotechnologies. Anti-ubiquitin antibody was obtained from Dako.

### Cytokine secretion

Cells were plated in 24-well plates and grown for 24 hours. They were serum-starved overnight and then maintained in culture for an additional 48 hours in the presence or absence of TNF. The supernatants were collected and the concentrations of IFN-γ, IL-1β, IL-2, IL-4, IL-5, IL-6, IL-8, IL-10 and IL-12 were determined by flow cytometry with the FlowCytomix human Th1/Th2 10plex bead kit and known standard curves, according to the manufacturer's instructions (Bender MedSystems). Beads were analyzed with a Beckman Coulter EPICS-XL MCL flow cytometer (Coulter Electronics Inc., Hialeah, FL) and data were analyzed with Bender MedSystems software. All the experiments were performed in triplicate. Intracellular cytokine production was studied in cells plated in six-well plates and grown for 24 hours. The cells were serum-starved overnight and stimulated for 16 h with TNF in the presence of brefeldin A (5 µg/ml). They were then lysed for analysis by western blotting.

### Transient transfection and reporter gene assays

The cells were plated in 24-well plates, grown to 50–80% confluence and transfected for 5 hours by the PEI (polyethylenimine) method, as previously reported [7]. For NF-κB luciferase reporter assays, we used 0.3 µg of NF-κB firefly luciferase reporter plasmid (Stratagene) per well. In some experiments, cells were transfected with the firefly luciferase reporter gene under the control of a full-length LCR (nucleotides 7464-37) from HPV5 (HPV5-flLCR/luc) (0.3 µg per well) or a minimal region of the LCR (nucleotides 189-37) from HPV5 (HPV5-minLCR/luc) (0.3 µg per well). The Renilla luciferase pRLTK vector (0.03 µg) was used as internal control for transfection efficiency. Luciferase activity was assessed with the Dual-Luciferase Reporter Assay kit (Promega) and a Centro XS^3^ LB 960 luminometer (Berthold Technologies). Data were normalized against protein concentration, as determined with the Bio–Rad protein assay (Bio-Rad). All the experiments were performed in triplicate.

For transient *EVER2* expression, cells were transfected with the *EVER2* cDNA encoding the wild-type EVER2 protein. The full-length Flag-tagged EVER2 expression vector was obtained as previously reported [7]. Gluc2-tagged TRAF1, TRAF2 and RIPK1 expression vectors were constructed by inserting cDNAs encoding human TRAF1 or TRAF2 into pSPICA-N2 vector containing a *Gaussia princeps* luciferase fragment (Gluc2), as previously described [38].

For transient siRNA transfection experiments, cells were transfected in the presence of INTERFERin™ (Polyplus transfection) ), according to the manufacturer's instructions.

### Semi-quantitative and quantitative real-time PCR

Total RNA was isolated with Trizol (GIBCO BRL). cDNA was produced by reverse transcription with the Superscript III First-Strand system (Invitrogen). The oligonucleotide sequences used for the semi-quantitative real-time PCR (RT-PCR) were, for *EVER2*: 5′-CTCTTCGGCACAGGAATTCGGTC-3′ and 5′-GCTGTACACGGAGCTGCTCTC-3′; and for *GAPDH*: 5′-GACCACAGTCCATGCCATCACT-3′ and 5′-TCCACCACCCTGTTGCTGTAG-3′.

### Cell fractionation and western blotting

Whole-cell extracts were prepared by incubating the cells for 45 minutes in lysis buffer (50 mM Tris-HCl, pH 7.4, 150 mM NaCl, 0.1% SDS, 0.5% DOC, 1 mM EDTA, 1% NP40, 1× complete protease inhibitor cocktail, 0.1 mM Na_3_VO_4_ and 10 mM NaF), on ice. For some experiments, nuclear and cytosolic protein fractions were separated with NE-PER Nuclear and Cytoplasmic Extraction Reagents (Pierce Biotechnology), according to the manufacturer's instructions. For EVER2 detection, insoluble fractions were prepared as previously described [27]. In some experiments, Triton-soluble and -insoluble fractions were prepared as previously reported [28]. The various fractions were subjected to electrophoresis and the protein bands were transferred to nitrocellulose membranes, which were probed with the appropriate antibodies and developed with the SuperSignal West Pico or West Femto Chemiluminescent Substrate System (Pierce Biotechnology). Lamin B and β actin were used as markers of the nuclear and cytoplasmic fractions, respectively. A Las-4000 image reader and Multi Gauge 3.1 software (Fuji Photo Film Co., Ltd.) were used for densitometric quantification of western blots. Data were normalized with respect to β actin for the cytoplasmic fraction and lamin B for nuclear fractions.

### Co-immunoprecipitation assay

Cells were lysed in immunoprecipitation buffer (50 mM Tris, pH 7.5, 150 mM NaCl, 1 mM EDTA, 0.5% NP40, 0.25% DOC, 1× complete protease inhibitor cocktail, 0.1 mM Na_3_VO_4_ and 10 mM NaF). Cell lysates were incubated overnight with 4 µg of antibody at 4°C. Complexes were precipitated with protein G/protein A-agarose (Calbiochem), washed and suspended in SDS sample buffer. Immunoprecipitates were subjected to SDS-PAGE and western blotting.

### Ubiquitination assay

For deubiquitination assays, HEK-293T cells were transfected with an HA-ubiquitin construct and other plasmids, as indicated. After 24 h, cells were stimulated with TNF for 2 h and harvested. Cell pellets were lysed as previously described [27]. The whole-cell extracts were subjected to immunoprecipitation and immunoblotting with the indicated antibodies. For endogenous TRAF2 ubiquitination assays, cells were plated and cultured for three days. They were then serum-starved for 6 h, harvested and lysed, as indicated above. Endogenous TRAF2 was immunoprecipitated with anti-TRAF2 antibody and subjected to immunoblotting with ubiquitin- and TRAF2-specific antibodies.

### Immunostaining and microscopy

Adherent cells were fixed with methanol and incubated with anti-p65 (Santa Cruz), anti-TRAF2 (Santa Cruz) or anti-KL1 (Immunotech Biotechnologies) and then with the Alexa Fluor 488–conjugated F(ab′)2 fragment of anti-mouse IgG (Invitrogen). The slides were mounted in VECTASHIELD Mounting Medium with DAPI (Vector Laboratories). Images were acquired with a Nikon Eclipse 80i fluorescence microscope (Nikon) with a 20× objective, a 377/482 nm filter and a Dxm 1200C digital camera (Nikon). They were analyzed with NIS Elements Imaging software (Nikon). The fluorescence intensity of nuclear p65 was quantified with ImageJ software (National Institutes of Health [NIH]).

Phase-contrast microscopy images were captured on a Nikon Microphot-FX microscope with a 20× objective.

### Statistics

The statistical significance of differences was determined with Student's *t* test. For all analyses, *P*<0.05 was considered significant.

## Supporting Information

Figure S1 **Schematic diagram illustrating the classical and alternative NF-κB signaling pathways, adapted from** [**32]**, **[39]**, **[40**] **.** Classical pathway involves activation of IKK complex by IKK-mediated IκBα phosphorylation, and subsequent degradation, resulting in nuclear translocation of the NF-κB heterodimer p65/p50. Alternative NF-κB pathway is dependent on NIK and IKKα and mediates the translocation of RelB/p52 complex. TNFR1 also activates JNK kinase, which activates AP-1 transcription factor.
(TIF)

Figure S2 **NF-κB and c-jun activation in the EV cell line.** EV and Healthy cell lines were left untreated or were stimulated with PMA+ionomycin (A) or TNF (B–E) for the times indicated. (A) Whole-cell lysates were analyzed by western blotting, to explore the IKKαβ complex. The phosphorylated forms of IKKαβ are indicated as phIKKαβ. The IKKα and actin bands on western blots were quantified by densitometry. Results are reported as the ratio of IKKα to actin. The ratio of the Healthy cell line at time 0 was set to 1. (B–C) Cytoplasmic and nuclear fractions were analyzed for the classical NF-κB and AP-1 axis (B) or the alternative NF-κB axis (C). The phosphorylated forms of p65, and c-jun are indicated as php65 and phc-jun, respectively. Whole-cell lysates (Total) were analyzed for total c-jun expression. The results shown are representative of three independent experiments. (D–E) Keratinocytes from the EV patient or the healthy subject were left untreated or were treated with TNF for 1 hour and stained for NF-κB (p65); the nucleus was stained with DAPI (D). Bars, 100 µm. (E) The fluorescence intensity of nuclear p65 was quantified before and after stimulation. Asterisks over error bars indicate statistically significant differences in fluorescence intensity between unstimulated and TNF-stimulated cells. Statistically significant differences between the two cell lines are indicated by asterisks over the brackets. Data are means ± SD of three independent experiments. *, *P*<0.05; **, *P*<0.01; ***, *P*<0.001.
(TIF)

Figure S3 **Expression of TRAF, p52 and c-jun in cells silenced for** ***EVER2*** **.** (A) miEVER2 and miCTRL cells were left untreated or were stimulated with TNF for the times indicated. Cytoplasmic extracts were subjected to western blot analysis. The results shown are representative of three independent experiments. (B) Healthy cells were transfected with siRNAs targeting various exons (5, 6, 8 and 10) of *EVER2* (siEVER2) or with a control siRNA (siCTRL) and were left unstimulated. Cytoplasmic and nuclear extracts or whole-cell lysates (Total) were subjected to western blotting. The results shown are representative of three independent experiments.
(TIF)
