# Supplementary figures and images for: Loss of the HPV-Infection Resistance EVER2 Protein Impairs NF-κB Signaling Pathways in Keratinocytes
Source: PLoS One. 2014 Feb 19;9(2):e89479. doi: 10.1371/journal.pone.0089479 (PMC3929693; doi:10.1371/journal.pone.0089479)

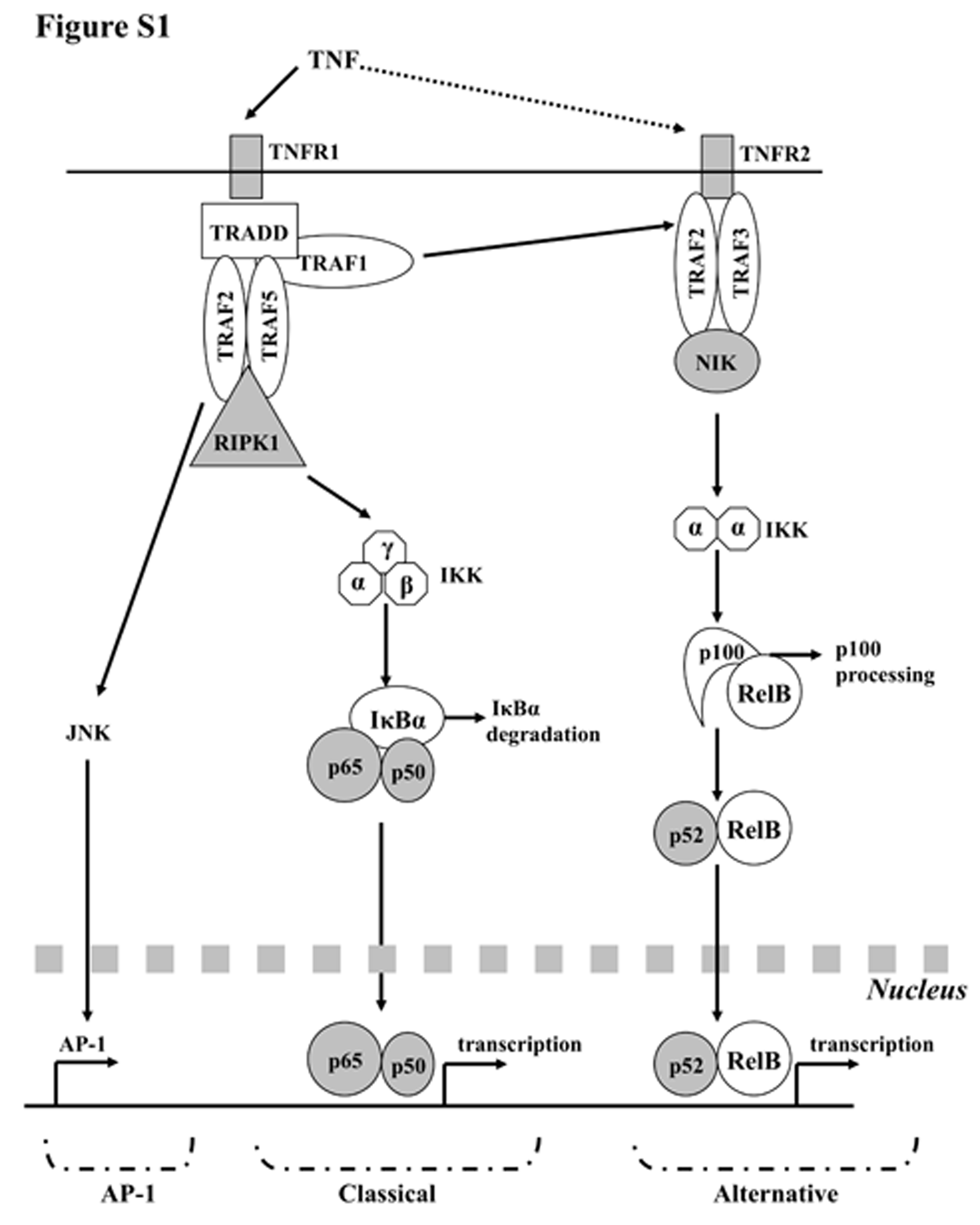

Supplement: Figure S1 — Schematic diagram illustrating the classical and alternative NF-κB signaling pathways, adapted from [32] , [39] , [40] . Classical pathway involves activation of IKK complex by IKK-mediated IκBα phosphorylation, and subsequent degradation, resulting in nuclear translocation of the NF-κB heterodimer p65/p50. Alternative NF-κB pathway is dependent on NIK and IKKα and mediates the translocation of RelB/p52 complex. TNFR1 also activates JNK kinase, which activates AP-1 transcription factor. (TIF) [file pone.0089479.s001.tif]

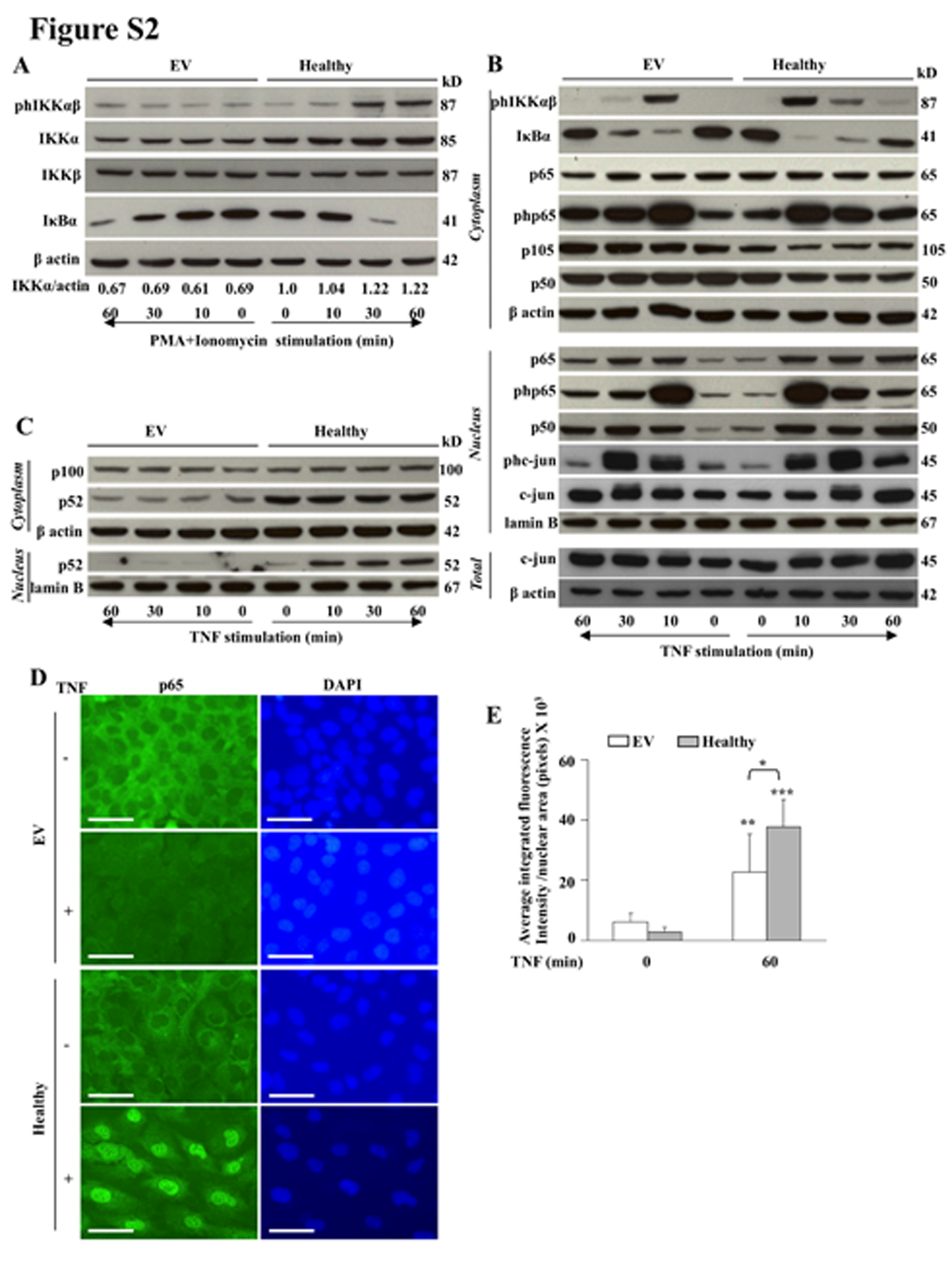

Supplement: Figure S2 — NF-κB and c-jun activation in the EV cell line. EV and Healthy cell lines were left untreated or were stimulated with PMA+ionomycin (A) or TNF (B–E) for the times indicated. (A) Whole-cell lysates were analyzed by western blotting, to explore the IKKαβ complex. The phosphorylated forms of IKKαβ are indicated as phIKKαβ. The IKKα and actin bands on western blots were quantified by densitometry. Results are reported as the ratio of IKKα to actin. The ratio of the Healthy cell line at time 0 was set to 1. (B–C) Cytoplasmic and nuclear fractions were analyzed for the classical NF-κB and AP-1 axis (B) or the alternative NF-κB axis (C). The phosphorylated forms of p65, and c-jun are indicated as php65 and phc-jun, respectively. Whole-cell lysates (Total) were analyzed for total c-jun expression. The results shown are representative of three independent experiments. (D–E) Keratinocytes from the EV patient or the healthy subject were left untreated or were treated with TNF for 1 hour and stained for NF-κB (p65); the nucleus was stained with DAPI (D). Bars, 100 µm. (E) The fluorescence intensity of nuclear p65 was quantified before and after stimulation. Asterisks over error bars indicate statistically significant differences in fluorescence intensity between unstimulated and TNF-stimulated cells. Statistically significant differences between the two cell lines are indicated by asterisks over the brackets. Data are means ± SD of three independent experiments. *, P<0.05; **, P<0.01; ***, P<0.001. (TIF) [file pone.0089479.s002.tif]

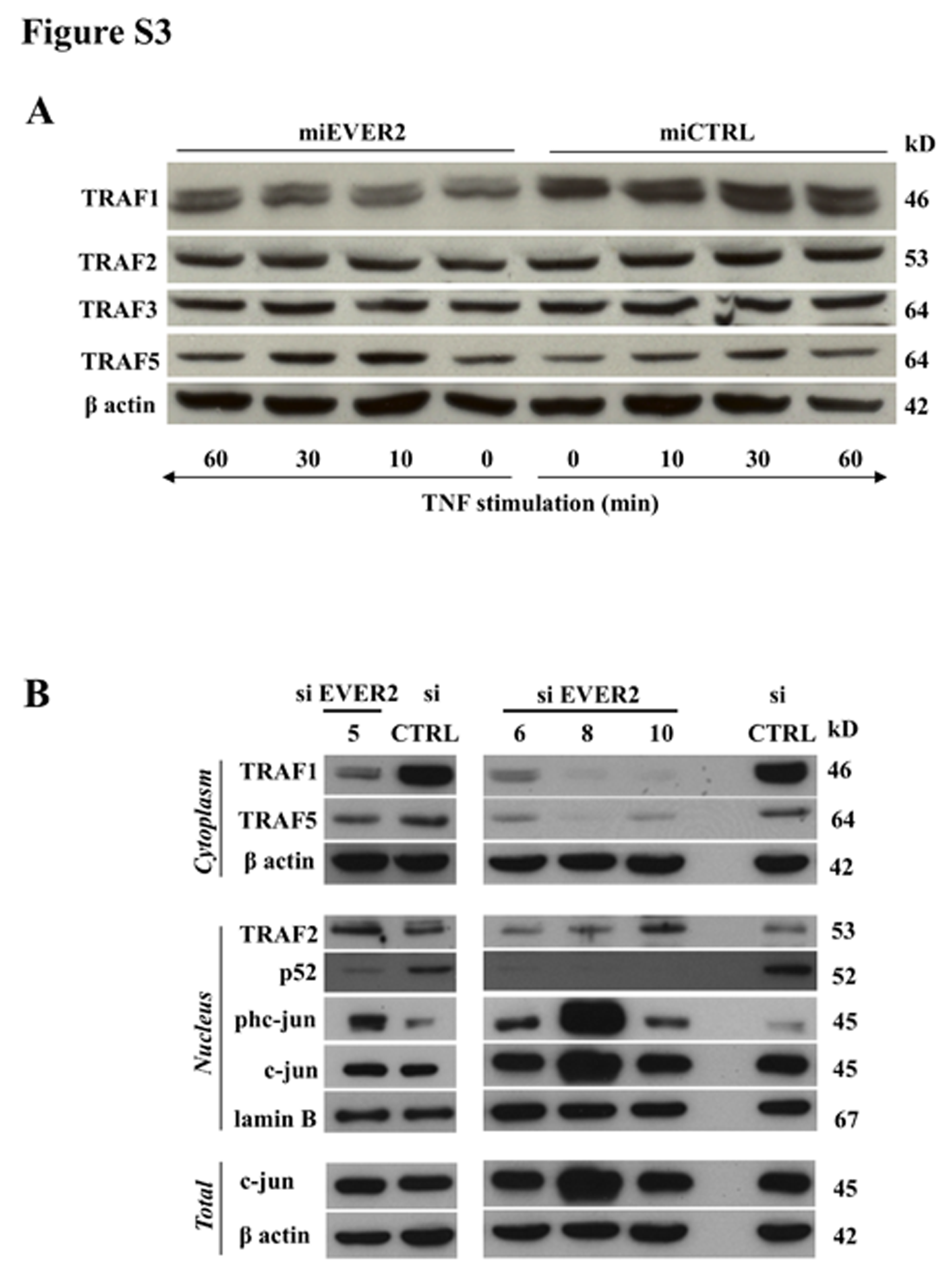

Supplement: Figure S3 — Expression of TRAF, p52 and c-jun in cells silenced for EVER2 . (A) miEVER2 and miCTRL cells were left untreated or were stimulated with TNF for the times indicated. Cytoplasmic extracts were subjected to western blot analysis. The results shown are representative of three independent experiments. (B) Healthy cells were transfected with siRNAs targeting various exons (5, 6, 8 and 10) of EVER2 (siEVER2) or with a control siRNA (siCTRL) and were left unstimulated. Cytoplasmic and nuclear extracts or whole-cell lysates (Total) were subjected to western blotting. The results shown are representative of three independent experiments. (TIF) [file pone.0089479.s003.tif]
